# Folate Conjugated Polyethylene Glycol Probe for Tumor-Targeted Drug Delivery of 5-Fluorouracil

**DOI:** 10.3390/molecules27061780

**Published:** 2022-03-09

**Authors:** Shabnam Sarwar, Muhammad Abdul Qadir, Rima D. Alharthy, Mahmood Ahmed, Saghir Ahmad, Michiel Vanmeert, Muhammad Usman Mirza, Abdul Hameed

**Affiliations:** 1 School of Chemistry, University of the Punjab, Lahore 54590, Pakistan; mabdulqadir@gmail.com (M.A.Q.); saghirtalib@gmail.com (S.A.); 2 Chemistry Department, Faculty of Science and Arts, King Abdulaziz University, Rabigh 21911, Saudi Arabia; 3 Department of Chemistry, Division of Science and Technology, University of Education, College Road, Lahore 54770, Pakistan; 4 Medicinal Chemistry, Department of Pharmaceutical and Pharmacological Sciences, Rega Institute for Medical Research, KU Leuven, B-3000 Leuven, Belgium; michiel.vanmeert@kuleuven.be (M.V.); umirzapk@gmail.com (M.U.M.); 5 Department of Chemistry and Biochemistry, University of Windsor, Windsor, ON N9B 3P4, Canada; 6 Department of Chemistry, University of Sahiwal, Sahiwal 57000, Pakistan; abdul_hameed8@hotmail.com

**Keywords:** folic acid, vector, HeLa cell, hepatoma mice, molecular dynamics simulations

## Abstract

A targeted delivery system is primarily intended to carry a potent anticancer drug to specific tumor sites within the bodily tissues. In the present study, a carrier system has been designed using folic acid (FA), bis-amine polyethylene glycol (PEG), and an anticancer drug, 5-fluorouracil (5-FU). FA and PEG were joined via an amide bond, and the resulting FA-PEG-NH_2_ was coupled to 5-FU producing folate-polyethylene glycol conjugated 5-fluorouracil (FA-PEG-5-FU). Spectroscopic techniques (UV-Vis, ^1^HNMR, FTIR, and HPLC) were used for the characterization of products. Prodrug (FA-PEG-5-FU) was analyzed for drug release profile (in vitro) up to 10 days and compared to a standard anticancer drug (5-FU). Folate conjugate was also analyzed to study its folate receptors (FR) mediated transport and in vitro cytotoxicity assays using HeLa cancer cells/Vero cells, respectively, and antitumor activity in tumor-bearing mice models. Folate conjugate showed steady drug release patterns and improved uptake in the HeLa cancer cells than Vero cells. Folate conjugate treated mice group showed smaller tumor volumes; specifically after the 15th day post-treatment, tumor sizes were decreased significantly compared to the standard drug group (5-FU). Molecular docking findings demonstrated importance of Trp138, Trp140, and Lys136 in the stabilization of flexible loop flanking the active site. The folic acid conjugated probe has shown the potential of targeted drug delivery and sustained release of anticancer drug to tumor lesions with intact antitumor efficacy.

## 1. Introduction

Traditional chemotherapy for cancer treatment has encountered a serious problem of no/slight specificity for cancer targets, eventually causing systemic toxicity. A promising method to resolve this issue is to design a specific delivery system to transport small molecules towards their target sites. Drug delivery systems along with a reduction in systemic toxicity exert significant treatment effects of attached antimicrobial and anticancer agents for infection and tumor [1,2]. Drug delivery probes labeled with radionuclides are capable of precisely highlighting the infection/tumor lesion in gamma cameras [3,4,5,6,7,8,9,10]. These tumor-targeting delivery systems have been extensively explored in previous decades, demonstrating that it is a promising methodology with reduced toxicity and selective chemotherapy [11,12,13,14,15,16,17]. Popular techniques for designing a cytotoxic agent’s delivery system to target cancer lesions include prodrugs with pH-sensitive polymeric coating or matrix formulations dependent on time [18,19,20,21].

A more recent and powerful approach is to target folate receptors (FOLRI). FLORI (FR) is defined as the receptors of glycosylphosphatidylinositol-anchored that exist on the cell’s surface and bind to folic acid with high affinity (Kd ≈ 0.1−1 nM) for its endocytosis. In a variety of cancers, FR receptors are overexpressed on the surface of cancerous cells, e.g., nasopharyngeal, breast, colorectal, renal, cervical, and ovarian cancers, compared normal cells [22,23]. FR receptors are present in three isoforms, namely FR-α, FR-β, and FR-γ, and the most overexpressed receptors are called FR-α. Contrary to resting macrophages, active macrophages possess FR receptors [24]. Folic acid (FA) has demonstrated a specific affinity for FR receptors that are over-expressed in cancer cells compared to normal ones. Therefore, FA-conjugated prodrugs are capable of differentiating among cancerous and normal cells [25]. To retain FA’s high affinity to FR receptors and to guarantee the successful delivery of anticancer drugs, the γ-carboxyl group of FA should be linked covalently [22,26,27,28,29]. As cancer progression and metastasis have increased the density of FR receptors and hence enhanced folate affinity for selective tumor targets [30,31,32,33]. Moreover, small-sized FA is suitable for developing several chemotherapeutic and antibacterial agents [34,35]. Folate-based targeted delivery of chemotherapeutic drugs has also been successfully applied for the imaging of tumors [3,36,37,38,39,40]. The critical point to design folic acid conjugate drug includes optimum constitution to penetrate inside cells and release the drug; i.e., the conjugate drug of folic acid contains folic acid, a cytotoxic agent, and a cleavable linker. Some folate conjugate drugs are already in clinical trials [41].

5-fluorouracil (5-FU) is a potent anticancer drug against various cancers such as breast, GI tract, and colorectal [42,43]. However, harmful side effects and poor target availability limit its clinical uses; i.e., the degradation of 5-FU in the digestive tract is detected with partial absorption [44,45]. It is believed that targeted drug delivery may overcome the limitations of 5-FU with increased efficacy at tumor lesions. A carrier system has attracted scientists due to the slow and sustained release of drugs through oral administration [46,47,48]. The literature revealed that biodegradable polymers are frequently explored as carriers; likewise, polyethylene glycol, pectin, dextrin, pullulan, azopolymers, and chitosan [49]. Amongst them, pegylation of drugs is considered a well-established technique to achieve enhanced biocompatibility, high solubility, and prolonged blood circulation [50,51,52]. FA is able to make binding with polyethylene glycol or cationic liposomes or polyethylene amine [29,53].

The presented work describes the design of a folic acid–PEG conjugate loaded with 5-FU, as well as its applications. The _ENREF_14_ENREF_14FA probe contains folic acid as a vector, polyethylene glycol bis-amine (linker b/w FA and 5-FU) as a prolonged serum circulator, and 5-FU as an anticancer drug. Synthesis includes a multi-step reaction: the first step involves the conjugation of folic acid to polyethylene-bis-amine (FA-PEG-NH_2_-), the second step includes 5-FU activation using formic acid (active methylene group), and third step includes the reaction of activated 5-FU with FA-PEG-NH_2_- to produce the final FA conjugate probe called FA-PEG-5-FU. FA conjugate was analyzed for drug release profile (in vitro drug release for 10 days) in PBS*,* cytotoxicity study in Vero cells; FR-affiliated uptake efficiency in HeLa cells, and targeted drug delivery at tumor lesion in artificial hepatoma mice models. To see the dynamics and binding potential of the prodrug, molecular docking study was performed, followed by molecular dynamic simulations. Later on, calculations of the molecular mechanics, binding energies, and surface areas (MM/GBSA) were rationalized to comprehend folic acid and folate conjugate interaction profile to FR receptors.

## 2. Results

**Figure 1:**
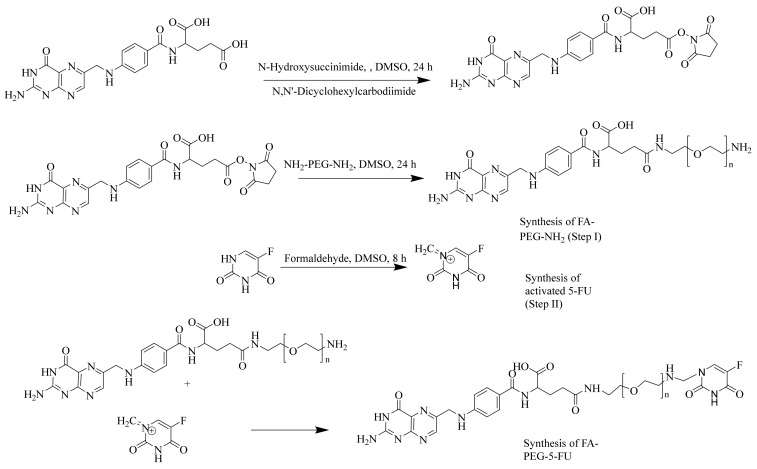
Synthesis of folate-conjugated, polyethylene glycol-loaded 5-fluorouracil.

Synthesis of FA-PEG-NH_2_ in Step 1m as shown in Figure 1, was accomplished using peptide bond formation as explained by Park et al. [54] (i.e., DCC coupling reaction). In Step 2, 5-FU was activated using formaldehyde in the presence of anhydrous DMSO to proceed with the reaction. Consequently, an active methylene group was introduced in the scaffold of 5-FU, which served as a linker among 5-FU and PEG amino units. UV-Vis, FTIR, and ^1^HNMR (see Supplementary Materials) characterization demonstrated the synthesis of prodrug (FA-PEG-5-FU) with desired purity and was also consistent with previously designed folate-based drug delivery carriers [26,55]. Figure 1 indicates that FA is linked to PEG-bisamine for the slow release of 5-FU.

### 2.1. In vitro Studies: Drug Release Profile, Cytotoxicity Assay, and Cellular Uptake Efficiency in FR Cells

**Figure 2:**
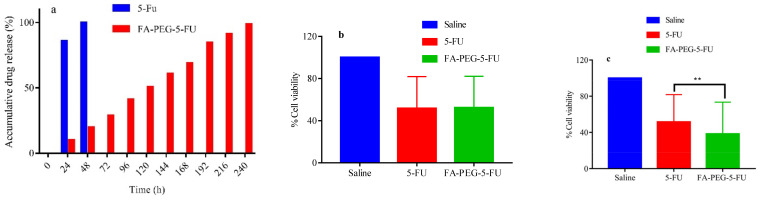
(**a**) Drug release profile, (**b**) in vitro cytotoxicity against Vero cells, (**c**) HeLa cells (significant difference at ** *p* < 0.005 5-FU vs. FA-PEG-5-FU).

Slow-release patterns of the drug in PBS (in vitro) showed a more near-linear profile for the release of 5-FU from FA-PEG-5-FU than to plain 5-FU over time; e.g., 5-FU is released fully (100%), as explained in Figure 2a. The folate conjugate probe was degraded slowly to release 5-FU in the buffer medium. The concentration of the free anticancer drug attained its maximum value in the first 48 h; however, prodrug showed only a 20% release. The 5-FU concentration after the 5th day increased up to 50% and 70% on the 7th day. The diffusion process was not involved only in the release of the drug; instead, it was supported by chemical control and depended on the hydrolyzation speed of the compound in the PBS, as explained in preceding studies [55]. The release pattern of the drug was analyzed using reverse-phase HPLC (Figure 2a).

The study of cytotoxicity assay against Vero cells showed a high mortality rate in the start in 5-FU treated cells; however, this rate decreased over time. Conversely, the cell inhibition rate in the test sample was slow at the beginning but increased over time. After 5 days of inoculation, prodrug (FA-PEG-5-FU) wells showed remarkable results compared to standard drug (5-FU) wells. These folate-conjugate-treated wells depicted slow and steady release of anticancer drug over time. The dose–response of the control, standard drug, and prodrug wells in Vero cells are presented in Figure 2b.

FR HeLa cells using MTT cell viability assay has demonstrated a noteworthy difference between standard drug (5-FU) and prodrug (FA-PEG-5-FU) wells, which may be considered due to the involvement of FR receptors facilitated endocytosis of folate conjugated (Figure 2c), which is consistent with previously reported studies [56,57].

**Figure 3:**
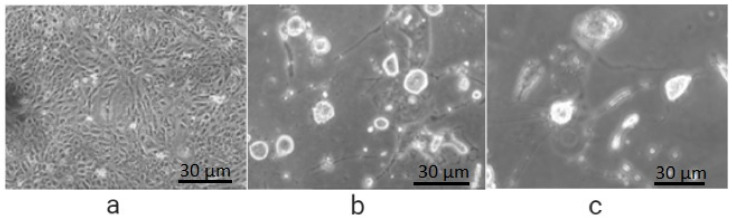
Cellular uptake efficiency in FR HeLa cells after 48 h of incubation: (**a**) with solvent (PBS) showing full growth; (**b**) treated with 5-FU, reduced growth; (**c**) treated with FA-PEG-5-FU showing less growth than 5-FU.

The encouraging results show that FA conjugation to PEG did not interrupt the affinity of folic acid to FR receptors (Figure 2c and Figure 3c). Furthermore, folate-conjugate-released anticancer drug inside the HeLa cells maintained its anticancer efficacy (Figure 2c and Figure 3c) as compared to standard (Figure 3b) [58].

### 2.2. Antitumor Efficacy in Mice Hepatoma Model

**Figure 4:**
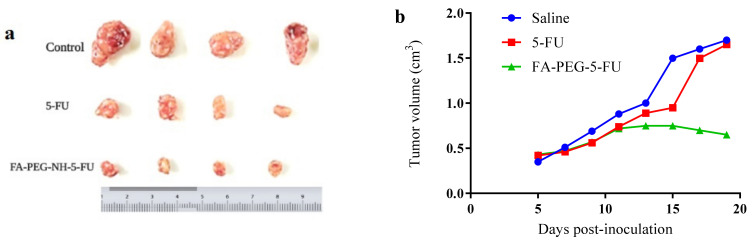
(**a**) Tumor sizes after administration; (**b**) tumor weights at 5, 10, 15, and 20 days post inoculation.

Hepatoma mice models showed that 15 min after prodrug (FA-PEG-5-FU) IV injection, the concentration of the conjugate was higher in plasma compared to other organs. Afterward, FA-PEG-5-FU plasma concentration decreased quickly and increased gradually after 24 h in the liver and spleen, attaining maximum values at 24 h. While at 72 h, the FA conjugate was found in the tumor cells, signifying long retention time than 5-FU, which was untraceable 6 h after its administration. The results indicate that the prodrug might release 5-FU slowly into the tumor cells and can exert an antitumor impact over an extended time period. Tumor sizes are given in Figure 4a, and the concentration of prodrug and 5-FU in the plasma vs. in the tumor is demonstrated in Figure 4b. The ratio of the initial concentration of 5-FU in the tumor vs. the blood was higher compared to that of FA-PEG-5-FU. Nevertheless 5-FU elimination at a greater rate limits its therapeutic effects. However, prodrug FA-PEG-5-FU retained the drug concentration in tumor cells for up to 7 days, regardless of some disparities that might be considered individual variances.

Data in Table 1 illustrate the index of organ to body weight. They show that the folate conjugate mice group showed lower weights of tumors than the standard mice group, suggesting the therapeutic efficacy of the prodrug [57,59].

### 2.3. Molecular Docking and Molecular Dynamics Simulations

**Figure 5:**
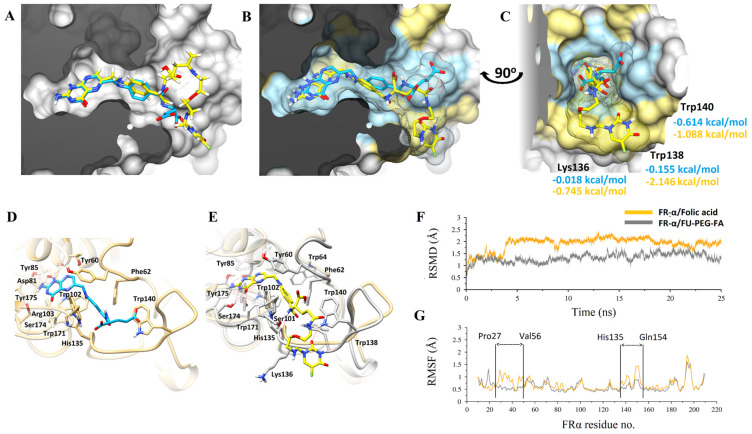
Molecular modeling studies of FA (in blue-grey sticks) and FA-PEG-5-FU (in yellow-orange sticks) in complex with FRα displayed in molecular surface representation. Molecular modeling studies of Folic acid (in blue sticks) and FU-PEG-FA (in yellow sticks) in complex with FRα displayed in molecular surface representation. (**A**), docked complexes with lowest energy ligand conformations are superimposed with surface clipped representation. (**B**) MD simulated stabilized conformations over a period of 25 ns are superimposed while the molecular surface of interacting residues is highlighted in blue (FA) and yellow (FU-PEG-FA), respectively. (**C**) Molecular surface in slab representation covering the deep binding groove is displayed with corresponding ligands in the same color scheme. The highly interacted residues after per-residue decomposition analysis are displayed with theoretical free binding energies. (**D**,**E**) FRα binding site residues are displayed interactively while the flexible flanking loop is more structured in (**E**). (**F**) Root-mean-square deviations (RMSD) for both complexes are plotted over a period of 25 ns while, (**G**) Root-mean square fluctuations (RMSF) with the flexible loops are pointed interactively.

Molecular modeling studies have elucidated the experimental findings reported above. In this work, the docking studies carried out generated a similar confirmation of binding, as displayed in Figure 5A–G. In prodrug (FA-PEG-5-FU), the FA moiety has shown a preserved pattern of theoretic binding of the co-crystalized complex of FA/FRα and interacts with similar residues, as reported previously [60]. An analysis of interaction was performed after folic acid prodrug docking with FR receptor and compared with native binding of FA-crystallized conformation with the receptor. The overall superimposition of each inhibitor to FRα is shown in Figure 5A. To obtain improved molecular insight into the stability of FA-PEG-5-FU/FRα and FA/FRα binding interactions, molecular dynamics (MD) simulations were executed over a 25 ns period in an explicit water atmosphere at constant pressure (1 atm) and temperature (300 K). The highlighted respective binding poses are presented in Figure 5A–C, revealing slab representation in the active site and surface mesh.

The analysis of the interaction of amino acid is presented in Figure 5D,E of FA/FRα and FA-PEG-5-FU/FRα, respectively. The prodrug (FA-PEG-5-FU) complex (gray), with the resultant MD simulation at 25 ns, is placed over FRα the intrinsic binding state (light brown), as seen in Figure 5D. The root-mean-square-deviation (RMSD) plot suggests additional FA/FRα elasticity than FA-PEG-5-FU/FRα (Figure 5F). RMSF provides further evidence wherever His135-Gln154 and Pro27-Val56 loops have more oscillations. The former fluctuating His135-Gln154 loop showed an enhanced structure in the FA-PEG-5-FU complex, as emphasized in Figure 5E, compared to FA/FRα (Figure 5G). The FA-PEG-5-FU complex showed diminished flexibility upstream compared to crystallized FA/FR complex (orange). The RMSD plot in Figure 5F illustrates conjunction of FA-PEG-5-FU and FA to FR^+^ receptors. Nevertheless, the complex FA-PEG-5-FU did not experience major conformational changes, signifying further stabilization effects as defined above.

Trp138, Trp140, and Lys136 amino acids of FA-PEG-5-FU complexes FRα have contributed to the loop-stabilizing effect as represented in Figure 5C. A flexible loop of His135-Gln154 amino acids is important in hydrogen bond stabilization of backbone and side chains’ interactions with glutamate [60]. The above-mentioned effect, along with the amino acids identification responsible for it, is rationalized through a free energy change calculation via decomposition analysis, RMSF, and RMSD calculations. Free energy changes, as shown in Figure 5C, from 0.614 to −1.088, −0.155 to −2.146, and −0.018 to −0.745 kcal/mol correspond to amino acids Trp140, Trp138, and Lys316. Comprehensive interaction analysis displayed that one hydrogen bond was donated from the 5-FU secondary amine to the oxygen backbone Trp138 carbonyl, and one hydrogen bond was also accepted from the Trp140 indole side chain. The nitrogen of the benzamide linker donated one hydrogen bond to the carbonyl backbone of HIS135. Assuming that FA-PEG-5-FU has larger size and flexibility, the scaffold-like pteridine is preferably positioned to make six hydrogen bonds to adjacent amino acids.

Total free binding energy of MM/GBSA is −65.2 and −52.73 kcal/mol for prodrug FA-PEG-5-FU and FA, respectively (Table 2). The total binding energy value is related to the number of hydrogen bonds and stability due to FU, suggesting higher electrostatic influences in the molecule of FA-PEG-5-FU (ΔE_ele_ = −89.92 kcal/mol) compared to FA (ΔE_ele_ = −79.25 kcal/mol).

Energy calculated in kcal/mol. ΔE_vdw_, van der Waals; ΔE_ele_, electrostatic; ΔE_MM_, molecular mechanics energy; polar (ΔG_p_) and non-polar (ΔG_np_) contributions; ΔG_sol_, solvation free energy; ΔG_to,_ total free binding energy

## 3. Discussion

Presented work demonstrates the binding FA-PEG-conjugated 5-FU and its facile movement through endocytosis in cancer cells with FR receptors and a preclinical study in tumor-bearing mice models. Studies revealed that small molecules of drugs are excreted rapidly through IV administration, which may lead to their non-specific distribution through capillaries inside normal tissues. Hence, systems are developed to deliver the drug to the target to decrease the side effects and enhance the therapeutic benefits of chemotherapy. FAs have been proved as targeting warheads for the specific delivery of imaging/therapeutic agents into cancerous cells. They act as active transport tools regardless of certain vitamin molecules’ absorption and cellular uptake. FAs have potential as drug delivery vectors if their in vivo recognition remains undisturbed after the pharmaceutical tethering [61,62]. At present, the mechanism of endocytosis of FA has been fully established; e.g., cancer cells FR receptors specifically recognize the FA moiety. FA’s low molecular weight makes it easy to be rapidly cleared through kidneys; thus, to prevent cellular excretion of the drug and enhance its in vivo lifetimes, PEGylation should be performed [63,64]. Therefore, we aim to develop a new conjugate of FA, PEG, and 5-FU and compare it to commercial 5-FU (unconjugated). Additionally, shielding by PEG can reduce toxicity and increment in circulation time [65]. Furthermore, polyplex PEG is able to reduce extracellular components’ interactions [66,67]. However, there are also certain disadvantages, such as reduction in cellular uptake and ineffective transfection [67]. Therefore, to make an effective strategy, ligands are added to PEG polyplexes and their transfection efficacy is optimized [68]. PEG’s main advantage is its facile conjugation with target agents, for example, folate [69] and galactose [70].

Here, long-chain bisamine (MW 3500–4500) was selected as a polymer linker between FA and 5-FU for its immunological and pharmacokinetic advantages [71]. PEG is an FDA-permitted agent used for PEGylation and is considered a standard for drug delivery systems’ solubility and bioavailability [52,72,73,74]. However, PEG efficacy of loading is significantly constrained due to two -OH groups [75]. Consequently, polyethylene glycol-bis-amine of higher molecular weight was the moiety of choice to make a covalent bond with a carboxylic acid of FA, resulting in an amide linkage. Direct coupling of 5-FU is not possible with PEG due to the -OH group deficiency in its structure. Accordingly, an active methylene group was introduced in the scaffold of 5-FU (activated). Active methylene group is able to make a covalent bond with the PEG terminal -NH_2_ group; thus, PEG bis-amine functions as a linker amongst 5-FU and FA. There are similar studies in the literature showing a folate conjugate of PEG-loaded 5-FU/Doxorubicin/Docetaxel/Adriamycin/Paclitaxel with successful delivery at the tumor lesion (Table 3).

The 5-FU was derivatized with FA for its targeted delivery at tumor lesions to avoid systemic toxicity and PEG to achieve better half-life and better tumor compliance [81]. The newly prepared compound bears FA, a vector molecule; 5-FU: an anticancer agent; and PEG, a polymer linker attached in such a way as not to disturb FA’s and 5-FU’s respective properties. FA conjugate was tested with a drug release profile study, anticancer properties, FR uptake efficacy, and antitumor activity in mice experimental models. The FA conjugate indicated a pattern of linear drug release overtime via a diffusion and hydrolysis mechanism, whereas 5-FU diffused in the PBS medium, with the PEG linker slowly degraded to release the anticancer agent. A continuous and steady drug-release profile was detected for ten days. The results have encouraged us to analyze the drug with a cytotoxicity study against Vero cells and FR uptake efficiency in HeLa cells to study the slow release profile, anticancerous activity, and FA-mediated endocytosis of 5-FU. The study of HeLa cells using an MTT cell viability assay illustrated an enhanced uptake of the prodrug in cancer cells compared to Vero cells, suggesting that FA cell permeation capability is integral and that amide linkage between PEG and FA has no influence on the FA-mediated targeting efficacy for FR cells. Additionally, results of the cytotoxicity assay (Vero and HeLa cells) also suggest that FA-PEG-5-FU conjugate slowly releases 5-FU with its intact anticancerous power.

In vivo studies of the folate prodrug (FA-PEG-5-FU) were conducted in murine hepatic cancer mice models, i.e., hepatoma mice models. These animal models are frequently employed to study the mechanisms of antitumor drugs [82]. Generally, it is believed that the development of tumors might be simulated in a better way than orthotopic tumor. Conversely, the complexity of the procedure and the high mortality rate made ectopic models the preferred ones. The mice were studied in groups: a positive control group (5-FU), a negative controls group (0.9% NaCl, Saline), and a test group (FA-PEG-5-FU). The comparison between the three groups illustrated that tumor volumes in two groups were decreased at the beginning of administrations, i.e., FA-PEG-5-FU and 5-FU groups, signifying enhanced antitumor activity compared to the negative control group. Tumor volumes of the control groups (5-FU and saline) increased considerably after the 15 days of the study, whereas mice in the test group showed a steady decrease in size, suggesting enhanced folate prodrug antitumor activity, which seems consistent with previous findings (drug release and anticancer activity studies). Consequently, the tumor volumes of the prodrug group (FA-PEG-5-FU) indicated a significantly smaller size of tumors compared to saline and 5-FU groups after 20 days of inoculation. To investigate the effects of tumor growth on the organ’s weight, we calculated the index of organ to body weight (o/b). The o/b index of the test group was found to be lower compared to the 5-FU and saline ones (Table 1). Therefore, in vivo results suggest that FA-PEG-5-FU maintained its FR-mediated endocytosis and cell penetration with 5-FU anticancer potential; i.e., folate conjugate transported the 5-FU at the tumor target, presenting long retention time, and released drug gradually within tumor lesion over time. Further studies are crucial to comprehend the folate conjugate’s full potential and to explore its pharmacokinetics as well as drug excretion mechanism.

## 4. Materials and Methods

### 4.1. Chemistry

Folic acid, formaldehyde, 5-fluorouracil, ethanol, chloroform, dimethyl sulfoxide (DMSO), methanol, Dulbecco’s phosphate-buffered saline (DPBS), neutral red, 3-(4,5-dimethylthialzol-2-yl)-2,5-diphenyltetrazolium bromide (MTT), hydrochloric acid, polyethylene glycol-bis-amine (MW 4000), *N*,*N*′-Dicyclohexylcarbodiimide, N-Hydroxysuccinimide, sodium hydroxide, and Glasgow minimum essential medium (GMEM, Invitrogen, Waltham, MA, USA) were purchased from Falcon Scientific, Lahore-Pakistan, originating from Sigma Aldrich, St. Louis, MO, USA, and all the reagents were of analytical grade. Water (0.01 μs/cm) of grade 1 quality was obtained from the apparatus available in the laboratory. Spectrometer NMR (Bruker, Billerica, MA, USA), ^1^HNMR (500 MHz), and FTIR spectrophotometer (Carry 630, Agilent Technologies, Santa Clara, CA, USA) were utilized to characterize products. Pre-coated German TLC (Merck, Kenilworth, NJ, USA) silica plates under UV light were employed to confirm the synthesis of new compounds with purity. A high-performance liquid chromatographic (HPLC) system, PG LC200, PC220 UV/Vis Detector, LC240 Vacuum Degasser, LC250 Column Oven, LC210 Pump, and Software (LC Win 1.0) fitted with Thermo Hypersil ODS column (C18, 4.6 nm × 250 mm, 5 μm) were employed to identify the new compound, its purification, and in vitro drug release.

### 4.2. Folate Prodrug (FA-PEG-5-FU) Synthesis

Folate prodrug synthesis includes three steps.

Step I: FA was reacted with PEG-bisamine to produce FA-PEG-NH_2_ as described below.

Folic acid (1.6 mmol, 0.784 g) was added to dissolve in DMSO (80 mL); then, to this solution, *N*,*N*′-Dicyclohexylcarbodiimide (1.8 mmol, 0.392 g) and Hydroxysuccinimide (1.8 mmol, 0.224 g) were added. The resulting mixture was whirled in darkness for the whole night. Dicyclohexyl urea, a by-product that appeared as white precipitates, was removed through vacuum filtration. Polyethylene glycol-bis-amine (M. W 4000 (0.1 mmol, 400 mg)) was added in a separate beaker to dissolve in 2 mL of DMSO. This solution was added to the above mixture obtained overnight. The whole mixture was swirled for 6 h in darkness until the product (FA-PEG-NH_2_) precipitates appeared, which were filtered and washed with methanol using Whatmann filter paper No. 42. The yield of the reaction was 68%. UV-Vis and ^1^HNMR were used for characterization of the product (FA-PEG-NH_2_), and spectral data are presented below.

UV-Vis (H_2_O, λ_max_): 280 nm; ^1^HNMR (D_2_O, 500 MHz): 11.06 (s, 1H, OH-carboxylic), 10.050 (s, 1H, NH-pyrimidine), 8.98 (s, 1H, -N=CH-), 8.03 (s, 1H, -COHN-), 7.59 (2H, d, *J* = 10.3, ArH), 6.85 (2H, d, *J* = 10.3, ArH), 4.59 (H, q, *J* = 7.5, CH), 4.35 (2H, d, *J* = 10.3, -CH_2_), 2.36 (t, 2H, d, *J* = 7.3, -CH_2_-succinimide), 2.08 (m, 2H).

Step II: To anhydrous DMSO (20 mL), 5-FU was added and heated at 55 °C with continuous stirring until dissolved, and then formaldehyde was added to it, followed by 8 h of stirring. An active methylene group was introduced in the scaffold of 5-FU at the end of the reaction.

Step III: The step-1 (FA-PEG-NH_2_) product was added to a beaker and dissolved in hot distilled water. This solution was added to the product solution from Step II (activated 5-FU) and stirred for 24 h until the appearance of precipitates of final folate conjugate (FA-PEG-5-FU). Physiochemical properties and spectral data are presented below.

Yield, 74.3%; *R_f_*, 0.82 (chloroform: methanol 80:20); UV-Vis λ_max_ (nm): 282, 365; IR υ_max_ (cm^–1^): 3246 (OH), 3338 (NH amide, stretching), 1014 (C-N amine), 1683 (N-H amide, bending), 1600, 1560 (C=C aromatic), 1768 (C=O carbonyl), 1122, 731 (C-H aromatic); ^1^HNMR (MeOD, 500 MHz): 11.34 (s, 1H, OH-carboxylic), 11.06 (s, 2H, NH-uracil), 10.050 (s, 1H, NH-pyrimidine), 8.98 (s, 1H, -N=CH-), 8.03 (s, 1H, -COHN-), 7.59 (2H, d, *J* = 10.3, ArH), 6.85 (2H, d, *J* = 10.3, ArH), 4.59 (H, q, *J* = 7.5, CH-methine), 4.35 (2H, d, *J* = 10.3, -CH_2_), 3.53 (2H, t, *J* = 7.3, -CH_2_-methylene-PEG), 3.34 (2H, t, *J* = 7.3, -CH_2_-methylene-PEG), 2.36 (t, 2H, d, *J* = 7.3, -CH_2_-methylene).

### 4.3. In Vitro Drug Release

5-FU (30 mg) and prodrug (equal to 30 mg 5-FU) were suspended separately at pH 7 in a phosphate saline buffer (PBS, 30 mL). In a dialysis bag, the above solution was transported, which was incubated at 37 °C. For quantitative analysis, compounds released in the medium were collected after every 24 h time interval from 24 h to 240 h and analyzed by HPLC.

### 4.4. Cytotoxicity Assays Study (In Vitro)

The cytotoxicity study of prodrug was performed in Vero cells using neutral red assay according to an established method [83,84] with slight modifications. Briefly, after fetal bovine serum (FBS, 10%) and Glasgow minimum essential medium (GMEM, Invitrogen, Waltham, MA, USA) were mixed, they were incubated (37 °C, 5% CO_2_) in a cell culture flask. All the apparatus used, including Eppendorf vials, micropipette tips, beakers, and well plates, was autoclaved prior to use. The dilutions of 5-FU (standard anticancer drug), PBS (control), and prodrug (FA-PEG-5-FU) were arranged (0, 10, 20, 30, 40, 50 μg/mL). All above-mentioned dilutions were shifted to 96-wells plate that already contained the following mixture: (i) GMEM medium (100 μL), (ii) FBS (10%), and (iii) cells monolayer (1 × 10^3^ cells/mL). Plates were incubated at 37 °C (72 h) under 5% CO_2_ conditions. After incubation time (72 h) and transferring of medium, the plates were rinsed using PBS without disturbance of the cells monolayer. Neutral red assay (NRD, 0.4 mg/mL), after dissolving with GMEM (200 µL), was added to well plates and incubated for 1 h. Afterward, the medium was decanted from the wells plates and washed using PBS for the elimination of detached dye. Those cells that absorbed dye were taken out using acidic ethanol (200 μL, 1% acetic acid) and incubated (10 min) to completely remove the dye. The solution of ethanol was measured for OD at 480 nm with a Microplate reader (BioRad, Hercules, CA, USA).

### 4.5. Cellular Uptake Efficiency in FR^+^ Cells

Folate conjugate’s targeting capability was analyzed using FR^+^ HeLa cells via MTT cell viability assay. In the 96-well plate’s culture medium, HeLa cells (1 × 10^3^ cells/well) were seeded and incubated for 48 h. Then, serial dilutions of 5-FU (standard anticancer drug), PBS (control), and FA-PEG-5-FU (prodrug, sample) were prepared (1, 5, 10, 25, 50 µg/mL) and added to the above-mentioned 96-well plate containing cancerous cells and then incubated for 72 h (37 °C). According to the ATCC manual, a freshly prepared MTT assay solution (5 mg/mL, 10 µL) was added in wells and incubated for 4 h. MTT was decanted, and DMSO (10 µL) was added to the residual solution. ELISA plate reader (BioRad, Hercules, CA, USA) was used for absorbance measurement at 650 nm, and cells viability (%) was calculated.

### 4.6. Antitumor Efficacy on Mice Hepatoma

Animals were divided into groups (*n* = 6) for in vivo studies, and for safety of animals, council directive (CEE 86/609) was adopted for our experiments, which was established by the EU on Animal Care and Experimentation, and the ethic committee of the School of Chemistry, University of the Punjab, Lahore-Pakistan has also approved it. Albino healthy mice (*n* = 24) of all sexes and approximately 3 months in age weighing between 20–25 g were purchased. During experiments, animal welfare was maintained and tested once a day, and all efforts were made to reduce the suffering of animals (living conditions with no pain). Standard conditions of housing were provided for all animals: 2 animals were housed per cage. Food and water were given in unlimited quantities to all animals. Regular monitoring of humidity and temperature was maintained. Animals were euthanized according to standard protocols at the end of the experiments.

Hepatoma cells (H22 cells, 2 × 10^6^) were subcutaneously injected in 24 mice’s left limbs to make hepatoma models. Mice without tumor signs after 72 h of inoculation were excluded. Mice bearing tumors were divided arbitrarily into three groups, and each group contained six mice (*n* = 6); mice from the 1st group (saline or negative control group) were injected with saline solution, mice of the 2nd group (5-FU or Positive control group) were injected with 5-FU, and mice of the 3rd group (FA-PEG-5-FU or Test group) were injected with Folate Prodrug. All mice groups were administered intravenously at 2 mg/Kg (at 5-FU equivalent) of their body weight. Tumor sizes of all mice groups were examined until 20 days and euthanized by the cervical cord dislocation after experiments. Organs comprising lungs, liver, heart, brain, spleen, kidney, and tumors were removed, washed with saline, and weighed. Then, calculations of tumor volumes, organ-to-body-weight index, and rate of tumor control were calculated by the following formula.

(1) $$\mathrm{Tumor}\;\mathrm{volume}\;=3/4\pi a^{2}\times b\;\mathrm{Organ}\;\mathrm{to}\;\mathrm{body}\;\mathrm{weight}\;\mathrm{index}\;=\;\mathrm{Weight}\;\mathrm{of}\;\mathrm{organ}/\mathrm{Weight}\;\mathrm{of}\;\mathrm{mouse}\;\times 100\;\mathrm{Tumor}\;\mathrm{control}\;\mathrm{rate}=\frac{\mathrm{Avg}.\;\mathrm{tumor}\;\mathrm{weight}\;\left(\mathrm{control};\mathrm{group}\right)-\mathrm{Avg}.\;\mathrm{tumor}\;\mathrm{weight}\;\left(\mathrm{test}\;\mathrm{group}\right)}{\mathrm{Avg}.\mathrm{tumor}\;\mathrm{weight}\;\left(\mathrm{control}\;\mathrm{group}\right)}$$

where *a* = length of the minor axis of tumor and *b* = length of the major axis of the tumor

### 4.7. Molecular Docking

Prodrug (FA-PEG-5-FU) studies for molecular docking were executed on the crystallized structure of folate receptor (PDB ID: 4LRH) complexes with folic acid [60]. Heteroatoms and water molecules were removed, and hydrogen atoms and charges were added to the target protein. Protein preparation, minimization, and optimization for docking details are the same as those previously described [85,86] employing Auto Dock Vina software (The Scripps Research Institute). The docking grid of FRα was recognized by covering the residues of the binding site of 24 Å × 24 Å × 24 Å in size. The protocol of applied docking was authenticated through redocking folic acid in a prescribed active site of crystallized structure. Then, docking of prodrug was performed in the explicit binding site, and those complexes with the highest binding affinity were considered for molecular dynamics simulations.

### 4.8. Molecular Dynamics Simulations

Molecular dynamics studies were carried out to understand the FRα binding dynamic consequences and FA and FA-PEG-5-FU stability inside the active site, according to the prescribed method [86,87]. In short, an ff99SB force field and general AMBER force field (gaff) were employed for FRα and inhibitors, respectively. The antechamber protocol, which was built in AmberTools16, was utilized for parameterization of docked structures. Use of leap module, produced coordinate files, and descriptive topology by considering residues at neutral pH value and their default protonation states. Sodium (Na^+^) and chloride ions (Cl^−^) were added prior to minimization of energy to neutralize the complex, centered in a simulation box of dodecahedral shape at a distance of 12 Å from the box edge, and solvated by water molecules (TIP3P). During MD simulations and MM minimization, long-range electrostatic interactions were treated using PME (particle mesh Ewald). Non-bonded interactions cut-off was set at 10 Å. A steepest descent minimization procedure was performed before MD simulations were employed to relax the complexes. Afterward, the system was heated from 0 K to 300 K over 150 ps in NVT ensemble along with 7.0  kcal/mol/Å2 restraint, and 1  ns NPT (T  =  300 K and P  =  1 atm) to perform MD simulations. Subsequently, all complexes at isobaric (1 atm) and isothermal (300 K) basis were exposed to 25 ns MD simulations. Hydrogen atoms were constrained via SHAKE at a 2.0 fs time period. Molecular Mechanics/Generalized Born Solvent Area (MM/GBSA) binding energy was used to attain rational insights into the difference of binding modes, and decomposition analysis based on MD simulations was carried out by investigating each residue binding’s contribution. The MM/GBSA technique realized in Amber16 was used to calculate the binding energies of inhibitors. The details of the MM-GBSA method have been explained in the literature [88,89]. MD trajectory was used to calculate all energy components between 5 and 25 ns. Total free binding energy (ΔG_tol_) is the sum of van der Waals (ΔE_vdw_) energy, molecular mechanics energy (ΔE_MM_), electrostatic (ΔE_ele_), and internal energy (ΔE_int_) in the gaseous phase. The solvation free energy (ΔG_sol_) is the sum of polar (ΔG_p_) energy and non-polar (ΔG_np_) energy as given below:

(2) $$∆E_{\mathrm{MM}}=∆E_{\mathrm{int}}+∆E_{\mathrm{ele}}+∆E_{\mathrm{vdw}}\;∆G_{\mathrm{sol}}=∆G_{p}+∆G_{\mathrm{np}}\;∆G_{\mathrm{tol}}=∆E_{\mathrm{MM}}+∆G_{\mathrm{sol}}$$

To calculate each residue’s contribution, MM/GBSA protocol of AMBER 16 [90] was carried out in the FRα binding pocket to the total free binding energy.

## 5. Conclusions

To prepare a folate conjugate probe for targeted drug delivery at cancer cells with gradual release of anticancer drugs, FA was conjugated to 5-FU by a bisamine-PEG linker. During in vitro studies of drug release profile and anticancer studies, prodrug (FA-PEG-5-FU) showed enhanced uptake in FR^+^ HeLa cells, along with considerably better bioavailability and increased retention time than 5-FU. Moreover, the present prodrug showed better in vivo antitumor efficiency with a prolonged half-life in mice. Molecular dynamics simulations have shown that amino acids Trp140, Trp138, and Lys136 stabilized the flexible loop of the active site, providing 5-FU and PEG comprehension on FR^+^ binding. Furthermore, the study offers awareness of FR^+^-influenced targeted drug delivery. Additional studies are necessary to analyze prodrug behavior in larger animals.

## Figures and Tables

**Table 1:** Index of organ to body weight (*n* = 6, mean ± SD).

Tissue	Saline	FA-PEG-5-FU	5-FU
Heart	0.4406 ± 0.34	0.4310 ± 0.43	0.4246 ± 0.41
Liver	5.5436 ± 0.27	6.0516 ± 1.03	5.3760 ± 0.96
Spleen	1.1261 ± 0.321	1.2616 ± 0.44	1.1976 ± 0.57
Lung	0.9816 ± 0.226	0.9156 ± 0.24	1.616 ± 0.50
Kidney	1.4616 ± 0.131	1.3177 ± 0.08	1.4361 ± 0.12
Brain	1.3561 ± 0.12	1.3161 ± 0.21	1.4116 ± 0.30
Thymus	0.1927 ± 0.08	0.2716 ± 0.07	0.2215 ± 0.15
Tumor	7.0817 ± 1.860	3.3917 ± 1.22	4.9476 ± 2.31

**Table 2:** Binding energies of FA and FA-PEG-5-FU.

	AD	ΔE_vdw_	ΔE_ele_	ΔE_MM_	ΔG_p_	ΔG_np_	ΔG_sol_	ΔG_tol_
FA	−7.1	−63.71	−79.25	−142.96	97.12	−6.89	90.23	−52.73
FA-PEG-5-FU	−9.6	−74.03	−89.92	−163.95	107.7	−8.95	98.75	−65.2

**Table 3:** Folate-based drug delivery carriers to transport anticancer agents at the targeted tumor lesion.

Drug Delivery Carriers	Drugs Loaded	Reference
Chitosan-PEG-Folate loaded 5-FU	5-Fluorouracil	[55]
Folate-poly(ethylene glycol)-poly(propylene succinate) nanoparticles	Paclitaxel	[76]
Folate-modified lipid-polymer hybrid nanoparticles	Paclitaxel	[77]
Folate-polyethyleneglycol-distearoylphosphatidylethanolamine (folate-PEG-DSPE) loaded DOX	Doxorubicin	[56]
Polymeric mixed micelles of poly(L-histidine)/PEG and poly(L-lactic acid) (PLLA) conjugate of folate	Adriamycin	[78]
Folic acid conjugated nanoparticles	Docetaxel	[79]
Folic acid-modified liposome-5-FU	5-Fluorouracil	[80]

## Data Availability

This study did not report any data.

## Supplementary Materials

The following supporting information can be downloaded at: https://www.mdpi.com/article/10.3390/molecules27061780/s1, Figure S1: 1HNMR: FA-PEG-NH_2_; Figure S2: 1HNMR: FA-PEG-5-FU.
