# Supplementary material for: Folate Conjugated Polyethylene Glycol Probe for Tumor-Targeted Drug Delivery of 5-Fluorouracil
Source: Molecules. 2022 Mar 9;27(6):1780. doi: 10.3390/molecules27061780 (PMC8954791; doi:10.3390/molecules27061780)
Supplement: Supplementary file 1 [file molecules-27-01780-s001.zip › molecules-1566774-supplementary.pdf]

Supplementary data

Folate Conjugated Polyethylene Glycol Probe for Tumor-Targeted Drug Delivery of 5-Fluorouracil

Shabnam Sarwar [1*], Muhammad Abdul Qadir [1], Rima D. Alharthy [2*] Mahmood Ahmed [3*], Saghir Ahmad [1], Michiel Vanmeert [4], Muhammad Usman Mirza [4], Abdul Hameed [5]

[1] School of Chemistry, University of the Punjab, Lahore-54590, Pakistan

[2] Chemistry Department, Faculty of Science and Arts, King Abdulaziz University, Rabigh 21911, Saudi Arabia

[3]Department of Chemistry, Division of Science and Technology, University of Education, College Road, Lahore-Pakistan

[4]REGA Institute for Medical Research, Medicinal Chemistry Herenstraat 49, 3000 Leuven Belgium

[6] Department of Chemistry, University of Sahiwal, Sahiwal-Pakistan

Running title: Folic acid assisted tumor-targeted delivery of 5- fluorouracil

To whom correspondence should be addressed

Shabnam Sarwar, PhD

shabnamshahzadkhan@gmail.com

Mahmood Ahmad, PhD

mahmoodresearchscholar@gmail.com, mahmood.ahmed@ue.edu.pk

Rima D. Alharthy, PhD

iaaalharte@kau.edu.sa

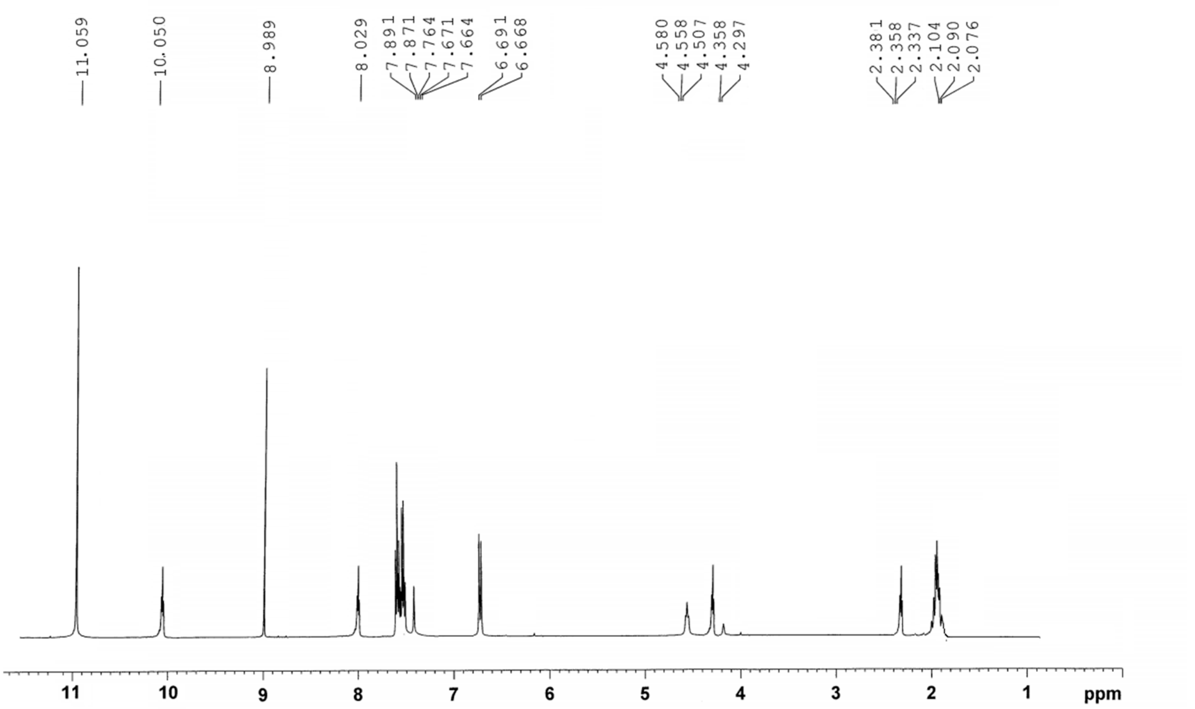

Fig. S1 ^{1}HNMR: FA-PEG-NH$_2$

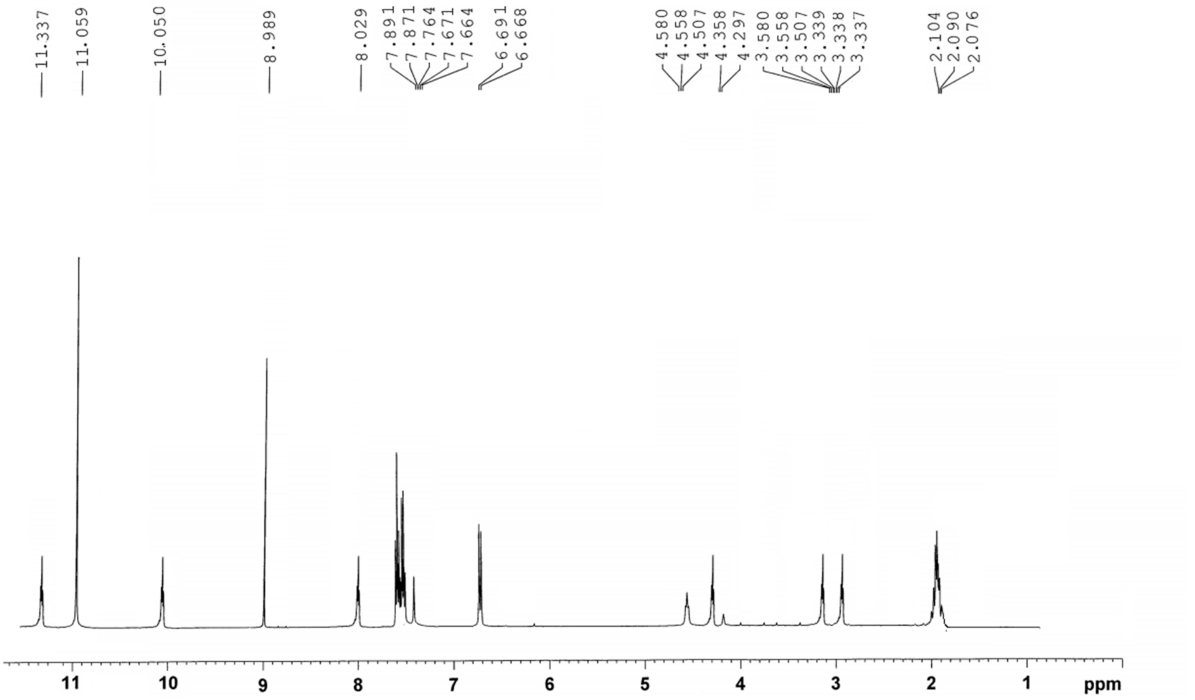

Figure S2 ^{1}HNMR: FA-PEG-5-FU
